# A Prospective Multicenter Assessment of the Accuracy and Safety of the Yuwell CT3 Real‐Time Continuous Glucose Monitoring System in Patients With Diabetes Over 14 Days

**DOI:** 10.1111/1753-0407.70246

**Published:** 2026-07-14

**Authors:** Lingli Zhou, Yufeng Li, Xingguang Zhang, Hongwei Ling, Yibing Lu, Linong Ji

**Affiliations:** ^1^ Department of Endocrinology and Metabolism Peking University People's Hospital Beijing China; ^2^ Department of Endocrinology Beijing Pinggu Hospital Beijing China; ^3^ Department of Endocrinology Army General Hospital of Chinese PLA Beijing China; ^4^ Department of Endocrinology The Affiliated Hospital of Xuzhou Medical University Xuzhou Jiangsu China; ^5^ Department of Endocrinology The Second Affiliated Hospital of Nanjing Medical University Nanjing China

**Keywords:** accuracy, continuous glucose monitoring, CT3, error grid analysis, MARD

## Abstract

- This 14‐day multicenter study assessed CT3 CGM accuracy and safety in 71 adults with diabetes.
- Key results: overall MARD 9.1% (median 7.1%), 82.4%–98.2% met %15/15–%30/30 criteria, 99.7% values in CEG zones A/B.
- CT3 showed strong performance, suitable for reliable 14‐day wear.

## Graphical abstract

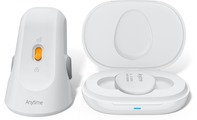

To the Editor.

Accurate and timely glucose monitoring is essential for diabetes management to prevent life‐threatening hypoglycemia and long‐term hyperglycemia‐related complications [1, 2], and to guide treatment adjustments [3]. Compared with intermittent self‐monitoring, real‐time continuous glucose monitoring (CGM) provides dynamic glucose profiles and detects otherwise unrecognized glycemic fluctuations [4]. The CT3 CGM system (Zhejiang POCTech Co. Ltd., Huzhou, China), developed by a company focused on biosensor‐based portable medical monitoring systems, is designed to improve signal stability and reduce electrochemical interference. It consists of an enzyme‐based sensor, a transmitter, and a mobile app (Figure S1). Interstitial glucose is oxidized by glucose oxidase, generating hydrogen peroxide that is electrochemically detected, with the resulting current proportional to glucose concentration. Data are transmitted via Bluetooth to a smart device, providing real‐time glucose readings and alerts for hypo‐ and hyperglycemia. The system supports flexible wear sites (upper arm or abdomen), is calibration‐free, and enables continuous use for up to 14 days. In this study, we evaluated its accuracy, safety, and alarm performance in adults with diabetes using venous blood glucose as the reference.

## 1 Methods

This prospective, multicenter, self‐controlled study (March–May 2022, five Chinese sites) enrolled 71 adults with type 1 or type 2 diabetes (T1D/T2D). Major inclusion criteria included age ≥ 18 years and willingness to wear the device for ≥ 14 days; key exclusions were skin diseases, allergies, bleeding tendency, pregnancy, or recent trial participation. A sample size ≥ 60 (per guidelines) ensured validity for primary outcomes [5]. Ethical approval (Peking University People's Hospital, No. 2021PHA165‐001) and informed consent were obtained.

Participants wore four CT3 sensors on bilateral upper arms and abdomen from day 1 and were randomly assigned to one 7‐h clinic session on Day 1/2, 7/8, or 14. During sessions, venous blood was collected every 15 min and measured using the Biosen Lactate and Glucose Analyzer (EKF Diagnostics GmbH, Leipzig, Germany), with each value paired to the nearest CGM reading within 5 min. Standardized meals were provided, and glucose levels were not intentionally manipulated. Insertion sites, adverse events, and usability were assessed on the final day.

Outcomes included accuracy (mean absolute relative difference [MARD], %15/15–%30/30, Clarke error grid [CEG], mean absolute difference [MAD], median absolute relative difference [ARD]) and hypo−/hyperglycemia detection and alert rates (sensitivity/specificity). Analyses were performed using R and SAS 9.4.

## 2 Results

All 71 participants (54.9% male, mean age 52.65 years [range 19–73]) completed the study; 22.5% had T1D and 77.5% had T2D (Table S1). A total of 8174 matched CGM‐EKF glucose pairs were obtained.

**FIGURE 1:**
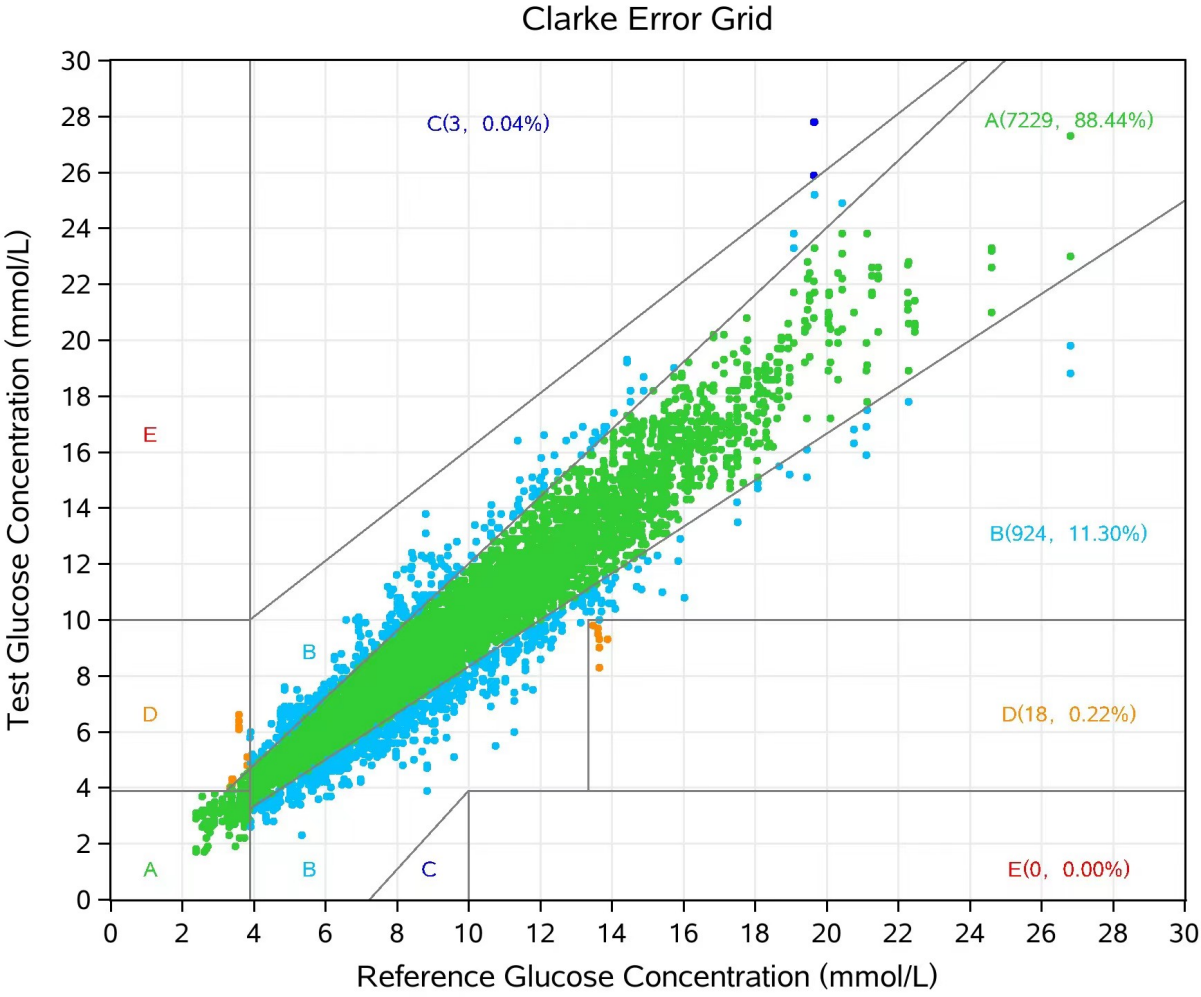
CEG analysis for CT3 system (*n* = 8174) compared with the reference venous blood glucose. CEG, Clarke error grid.

Overall MARD was 9.1% (median ARD 7.1%), with agreement rates of 82.4% (15/15), 91.7% (20/20), and 98.2% (30/30). Performance was comparable between arm and abdomen sites (both MARD 9.1%) (Table 1). Among sensors, 64.6% showed MARD < 10%, while 9.1% exceeded 20%. CEG analysis showed 99.74% of all values in zones A/B (Figure 1).

**TABLE 1:** Overall MARD and agreement rates.

Placement	Matched pairs (*n*)	%15/15 (%)	%20/20 (%)	%30/30 (%)	MARD (%)	Median ARD (%) (IQR)
Overall (*N* = 71)	8174	82.4	91.7	98.2	9.1	7.1 (3.2, 12.7)
Arm (*N* = 71)	4087	82.1	91.8	98.2	9.1	7.2 (3.1, 12.9)
Abdomen (*N* = 71)	4087	82.7	91.7	98.2	9.1	7.1 (3.3, 12.6)

Abbreviations: ARD, absolute relative difference; IQR, interquartile range; MARD, mean absolute relative difference.

In the target glucose range (80–180 mg/dL), MARD was 9.5%–9.7%; mild‐to‐moderate hyperglycemia (181–300 mg/dL) showed MARD of 7.8%–8.1%, and severe hyperglycemia (301–400 mg/dL) 7.5%–8.1%. In hypoglycemia (40–80 mg/dL), MAD was 6.4–8.3 mg/dL (Table S2). For rapid glucose changes (< −2 or > 2 mg/dL/min), accuracy declined (arm MARD: 16.2%–16.3%; abdomen: 16.4%–16.6%) (Table S3).

Hypoglycemia detection rates were 83.3% (arm) and 77.8% (abdomen), with corresponding alert accuracies of 97.1% and 97.4%. Hyperglycemia detection rates were 81.8% (arm) and 78.8% (abdomen), with alert accuracies of 96.9% and 91.7%.

Sensor survival probability was 99.65%, with 70/71 participants using sensors for ≥ 14 days without abnormality. No significant device‐related adverse events were reported. One participant had minor insertion‐site reactions (suspected device‐related); one unrelated diabetic ketoacidosis occurred. Overall, 98.6% of participants found the device comfortable; 59.2% rated it “outstanding” and 40.9% “satisfactory.”

## 3 Discussion

This study demonstrates that the CT3 CGM system provides accurate, safe, and reliable glucose monitoring over 14 days in Chinese adults with diabetes, offering important insights for clinical application in a population with high diabetes burden [6]. The system showed strong overall accuracy, with MARD 9.1%, compared with reported values of 14.8% for FreeStyle Libre Pro [7] and 13.6% for Dexcom G7 [8]. Over 64.5% of sensors showed single‐digit MARD in non‐hypoglycemic ranges, with consistent accuracy across arm and abdominal sites.

Alert accuracy was high (88.80% for hypoglycemia and 94.71% for hyperglycemia), although lower accuracy in isolated cases warrants further investigation. While overall MARD may not fully reflect system accuracy, CT3 showed minimal rate‐of‐change dependence and high agreement in CEG (99.74% in zone A/B) [9], comparable to reported values for Dexcom G6 (98.7%) [10] and Abbott Freestyle Libre 1 (99.8%) [11]. The 80 mg/dL hypoglycemia alert threshold, consistent with CLSI POCT05 recommendation (70–100 mg/dL) [12], enabled timely detection and may support earlier intervention, reducing time in hypoglycemia [13]. Questionnaire results indicated high user compliance and favorable perceptions of usability and real‐time alerts, although no significant quality‐of‐life improvement was noted. Safety was confirmed, with no severe adverse events; minor insertion‐site reactions were infrequent.

Limitations include a modest sample size that may be unrepresentative of subgroups, absence of clinical outcome assessments, and potential statistical bias. In addition, participants were not stratified by treatment regimen despite collection of concomitant medication data, which may have influenced glycemic variability and CGM accuracy. The overrepresentation of T2D relative to T1D may also limit generalizability. Future studies should include larger, more diverse cohorts, particularly patients with T1D and pediatric populations, to further evaluate CGM performance.

## Funding

This work was supported by National Science and Technology Major Project (Grant No. 2024ZD0530204).

## Conflicts of Interest

The authors declare no conflicts of interest.

## Supporting information


**Figure S1:** CT3 system components: transmitter (left) and wearable sensor (right).
**Table S1:** Baseline demographics of study participa (*n* = 71).
**Table S2:** Accuracy performance at different reference glucose levels.
**Table S3:** Accuracy performance at different rates of change in glucose level.

## Data Availability

The data that support the findings of this study are available from the corresponding author upon reasonable request.
