# Supplementary material for: A Prospective Multicenter Assessment of the Accuracy and Safety of the Yuwell CT3 Real‐Time Continuous Glucose Monitoring System in Patients With Diabetes Over 14 Days
Source: J Diabetes. 2026 Jul 14;18(7):e70246. doi: 10.1111/1753-0407.70246 (PMC13366774; doi:10.1111/1753-0407.70246)
Supplement: Supplementary file 1 — Figure S1: CT3 system components: transmitter (left) and wearable sensor (right). Table S1: Baseline demographics of study participa (n = 71). Table S2: Accuracy performance at different reference glucose levels. Table S3: Accuracy performance at different rates of change in glucose level. [file JDB-18-e70246-s001.docx]

**Supplementary Material**

**
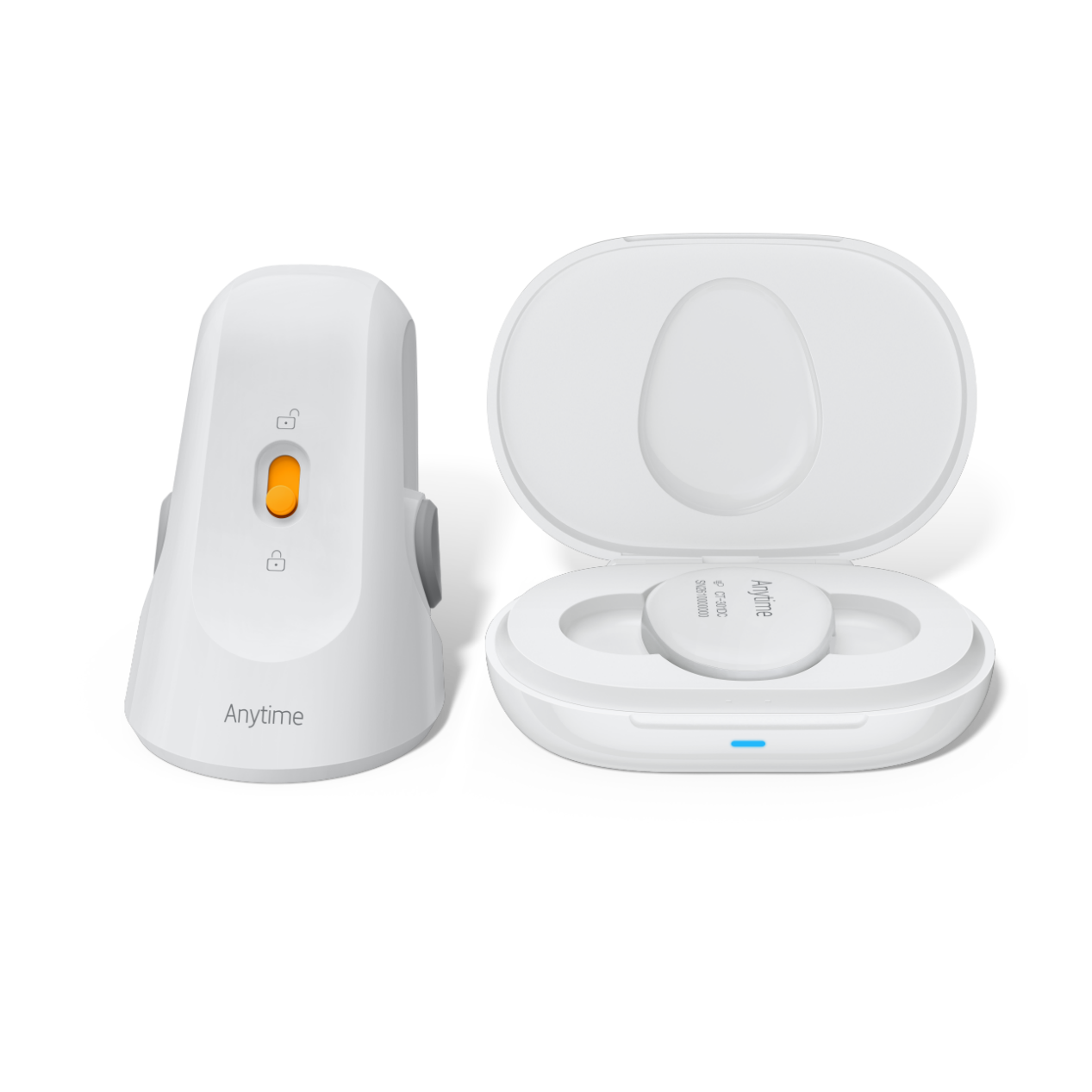
**

**Supplementary Figure 1. CT3** **system components: transmitter (left) and wearable sensor (right).**

**Supplementary Table 1. Baseline demographics of study participants (n = 71)**

| *Demographics* | *Combined (n = 71)* |
| --- | --- |
| Age, years, Mean (SD) | 52.65 (13.4) |
| Sex, n (%) |  |
| Male | 39 (54.9) |
| Female | 32 (45.1) |
| BMI category, n (%) |  |
| Underweight (<18.5kg/m^2^) | 2 (2.8) |
| Normal (18.5 to <25kg/m^2^) | 34 (47.9) |
| Overweight (25 to <30kg/m^2^) | 25 (35.2) |
| Obese (≥30kg/m^2^) | 10 (14.1) |
| BMI, kg/m^2^ |  |
| Mean (SD) | 26.9 (8.8) |
| Median | 24.8 |
| Min, Max | 17.3,62.5 |
| Ethnicity, n (%) |  |
| Han ethnic group | 68 (95.8) |
| Man ethnic minority | 2 (2.8) |
| Hui ethnic minority | 1 (1.4) |
| Diagnosis, n (%) |  |
| Type 1 diabetes | 16 (22.5) |
| Type 2 diabetes | 55 (77.5) |
| Medical history, n (%) | 42 (59.2) |
| Concomitant medication history, n (%) | 70 (98.6) |

*Abbreviations: BMI, body mass index; SD, standard deviation*

**Supplementary Table 2. Accuracy performance at different reference glucose levels**

| *Placement* | *Glucose range*  *(mg/dL)* | *Matched*  *Pairs (n)* | *%15/15*  *(%)* | *%20/20*  *(%)* | *%30/30*  *(%)* | *MAD*  *(mg/dL)* | *MARD*  *(%)* |
| --- | --- | --- | --- | --- | --- | --- | --- |
| *Arm (N=71)* | (40, 60] | 38 | 89.5 | 97.4 | 100.0 | 6.4 | NA |
|  | (60, 80] | 166 | 86.7 | 95.8 | 98.8 | 8.3 | NA |
|  | (80, 180] | 2349 | 78.2 | 89.2 | 97.2 | NA | 9.7 |
|  | (180, 300] | 1402 | 86.5 | 94.5 | 99.4 | NA | 7.8 |
|  | (300, 400] | 122 | 89.3 | 95.9 | 99.2 | NA | 7.5 |
|  | (400, Inf] | 10 | 70.0 | 80.0 | 100.0 | NA | 11.6 |
| *Abdomen (N=71)* | (40, 60] | 38 | 94.7 | 100.0 | 100.0 | 7.0 | NA |
|  | (60, 80] | 166 | 86.1 | 92.2 | 97.6 | 8.0 | NA |
|  | (80, 180] | 2378 | 79.7 | 90.0 | 97.7 | NA | 9.5 |
|  | (180, 300] | 1380 | 86.2 | 93.5 | 98.9 | NA | 8.1 |
|  | (300, 400] | 115 | 83.5 | 93.0 | 98.3 | NA | 8.1 |
|  | (400, Inf] | 10 | 90.0 | 90.0 | 100.0 | NA | 8.0 |

*Note: MAD is calculated in hypoglycemia ranges (≤ 80mg/dL), while MARD for ranges >80mg/dL*

*Abbreviations: MAD, mean absolute difference; MARD, mean absolute relative difference; NA, not applicable*

**Supplementary Table 3. Accuracy performance at different rates of change in glucose level**

| *Placement* | *CGM rate of change (mg/dL/min)* | *Matched pairs (n)* | *%20/20 (%)* | *MARD (%)* |
| --- | --- | --- | --- | --- |
| Arm (N=71) | <-2 | 124 | 33.87 | 16.2 |
|  | -2 to <-1 | 407 | 78.62 | 13.3 |
|  | -1 to <0 | 1,357 | 99.34 | 5.4 |
|  | 0 to <1 | 1,492 | 99.20 | 5.3 |
|  | >1 to 2 | 534 | 84.64 | 12.9 |
|  | >2 | 173 | 43.35 | 16.3 |
| Abdomen (N=71) | <-2 | 116 | 36.21 | 16.4 |
|  | -2 to <-1 | 383 | 77.02 | 13.1 |
|  | -1 to <0 | 1,415 | 99.22 | 5.6 |
|  | 0 to <1 | 1,469 | 99.66 | 5.3 |
|  | >1 to 2 | 542 | 84.32 | 12.9 |
|  | >2 | 162 | 34.57 | 16.6 |

*Abbreviations: MARD, mean absolute relative difference*
